# The Medical Profession

**Published:** 1899-09-02

**Authors:** 


### The Hospital

A Journal of

The Medical Sciences and Hospital Administration.

Vol. XXVI.—No. 675.] Edited by Sir Henry Burdett, K.C.B., and Solomon C. Smith, M.D., M.R.C.P. [September 2, 1899.

### The Medical Profession.

The appearance of our "Educational Number" indicates the near approach of the day on which large additions are annually made to the ranks of those who are preparing to enter the medical profession, and on which some who have taken the first step in this direction in a previous year may yet feel tempted to pause, and to consider how far their experience has demonstrated their fitness for the calling which, in comparative ignorance of its requirements, they have been induced to select. The aspects of medicine are various, and appeal to many different forms of taste and energy. We are told in many directions that the profession is overcrowded; but we have been told the same thing, not of the medical profession only, but of many others, for so long a time that the memory of man runneth not to the contrary. The overcrowding was much dwelt upon in the House of Commons during the debates which led to the passing of the Medical Act of 1858. Nevertheless, the cry is still "they come." During the intervening forty years the number of doctors in the United Kingdom has increased enormously, notwithstanding increased requirements in the way of study and examination, and the increased cost which these requirements entail. But the population has increased, perhaps in about the same ratio, wealth has increased, certainly in a still greater ratio, and the progress of science has multiplied alike the opportunities for research and the opportunities for usefulness, the power of acquiring knowledge and the power of applying it. There is less room than formerly, very possibly, for laziness and ineptitude; but it will always be open to those who are so gifted to become medical reformers, and to take the chestnut of notoriety out of the embers for the benefit of persons more astute and less honest than themselves. But, wherever there is an ambition to become, before all other things, a good doctor, the student will soon find that the one object of medical work is to discover and establish what is true, and then to apply the truth to the preservation of life and to the relief of suffering. In this respect medicine stands alone. The professional politician lives in bondage to fools, and must render their folly articulate as a condition of obtaining their suffrages. The advocate must often feel that the establishment of truth would be fatal to his contention. The theologian must often glide swiftly over the thin places of his argument. The advertiser, to quote words used by Bishop Earle, "tells lies trippingly and by rote, as the proper language to sell in, and that which he has spent most of his life to learn." Even the supposed philosopher does not always "love truth better than his system," and does not always turn an impartial ear to arguments destructive of his hypothesis. To the doctor everything which is not true is useless. His knowledge, although constantly expanding, is still confined within narrow limits; but, within those limits, it is trustworthy and complete. Like other men, he is often compelled to guide his actions by high probability, because, even in the presence of doubt, he is still called upon to *do;* but he need not mistake probability for demonstration, and he should not be even able, still less should he be prone, to conceal his own ignorance from himself. He is perpetually "wanting to know," and, whether he devote himself personally to research, or is content to assimilate the results of the research of others, the conditions of his utility are the same. If it be true that a given cause will produce given consequences, then he can confidently proceed, in fitting circumstances, to bring this cause into operation for the attainment of his ends. If the connection between the presumed cause and the presumed effect be doubtful, or if it be dependent upon conditions which are wholly or partially unknown, medical action is so far rendered halting and uncertain. With a possible exception in favour of those furnished by the higher mathematics, the doctor is brought into daily contact with the most difficult of all problems; problems in which the most recondite facts of physical or physiological science are complicated by the ever-varying physical and moral conditions of the human patient. Much of his work is of necessity empirical in the strict sense; that is to say, it is based upon experience of what has happened in similar circumstances on other occasions, and without full knowledge of all the factors by which the ultimate result was brought about. By some men, perhaps by many, this empirical knowledge will be accepted as sufficient; by others, it will be accepted only provisionally, and used chiefly as a stimulus to research. Where adequate mental capacity does not exist, no education can confer the power of weighing evidence, the power of correct observation, the power of drawing sound inferences, the power of maintaining a suspended judgment, although without arrest of needful activity, when the evidence is insufficient to justify an intellectual conclusion. Education can, however, do much to arouse whatever capabilities of these kinds may be dormant in the learner; and, inasmuch as the discovery and establishment of truth are the highest functions of which the human mind is capable, the functions which differentiate a Newton or a Faraday from the many persons who argue without being able to reason, so it is of the highest importance that medical education, throughout its whole course, should be so directed as to develop to the fullest possible extent whatever qualities, in the direction indicated above, the pupil may be so fortunate as to possess. And, when the education has been completed, the student enters upon a career superior to all others alike in interest and in variety. In an empire on which the sun never sets, there is no place and no day in which the members of the medical profession may not be found at the post of duty, wherever this may be, in the palaces of the wealthy, in the slums of cities, in the lonely hamlet, in the beleaguered garrison, in the tented field. No other calling affords equal opportunities of observing and studying the essentials, as distinguished from the accidentals, of human nature; no other is calculated so to entwine the love of truth with every fibre of the intellectual organism, and no other affords even approximately equal opportunities of doing good.

